# Increasing planting density can improve the yield of Tartary buckwheat

**DOI:** 10.3389/fpls.2023.1313181

**Published:** 2023-12-13

**Authors:** Qiuyue Zhou, Peiyun He, Jingang Tang, Kaifeng Huang, Xiaoyan Huang

**Affiliations:** ^1^ School of Life Science, Guizhou Normal University, Guiyang, China; ^2^ Guizhou Institute of Mountain Resources, Guizhou Academy of Sciences, Guiyang, China

**Keywords:** planting density, Tartary buckwheat, low-nitrogen, senescence, yield

## Abstract

Planting densities and nitrogen fertilizer application rates determine the yield of crops. Tartary buckwheat is a pseudocereal crop with great health care and development values. However, little is known about application of nitrogen fertilizer and planting density on the physiological characteristics of Tartary buckwheat. This study aims to clarify the effect of planting density on the senescence and yield of Tartary buckwheat under low nitrogen conditions. A 2-year field experiment was conducted on Tartary buckwheat (Jinqiao 2) to study the effects of different planting densities (8 × 10^5^, 10 × 10^5^, 12 × 10^5^, 14 × 10^5^, and 16 × 10^5^ plants·ha^−1^) on the root morphology and activity, chlorophyll and malondialdehyde (MDA) contents, antioxidant enzyme activity, photosynthetic characteristics, agronomic traits, and yield of Tartary buckwheat in the absence of nitrogen fertilizer treatment. With the increase in planting density, the root morphological indices and activities; chlorophyll a, chlorophyll b, and carotenoid contents; superoxide dismutase and peroxidase activities; net photosynthetic rate; transpiration rate; intercellular CO_2_ concentration and transpiration rate; main stem node, branch, and leaf numbers; grain number and weight per plant; and 1000-grain weight of Jinqiao 2 decreased continuously, whereas plant height and leaf MDA content increased continuously. The yield of Tartary buckwheat first increased and then decreased with the increase in planting density. The yield under 14 × 10^5^ plants·ha^−1^ treatment increased by 68.61%, 44.82%, 11.00%, and 22.36%, respectively, relative to that under 8 × 10^5^, 10 × 10^5^, 12 × 10^5^, and 16 × 10^5^ plants·ha^−1^treatments. In summary, planting at an appropriately high density (14 × 10^5^ plants·ha^−1^) can promote the increase in the yield of Tartary buckwheat populations under low nitrogen conditions and is recommended for use in production to achieve the high-yielding and nitrogen saving cultivation of Tartary buckwheat. This research can serve as a theoretical basis to jointly achieve the high yield and nitrogen saving of Tartary buckwheat.

## 1 Introduction

Buckwheat (*Fagopyrum*) is a member of genus *Fagopyrum* Mill. It has three main cultivated species, namely, common buckwheat (*Fagopyrum esculentum* Moench), Tartary buckwheat (*Fagopyrum tataricum* Gaertn), and golden buckwheat (*Fagopyrum cymosum*) (Zhang et al., 2023a). China is the origin of buckwheat and has a long history of buckwheat cultivation (Zhang et al., 2021a). Tartary buckwheat is rich in flavonoids (rutin and quercetin), D-chiral inositol, and active polysaccharides and thus has health care value. It can reduce blood lipid and glucose levels and decrease and alleviate the occurrence of scurvy, cancer, and other major diseases (Steadman et al., 2001; Wang Y. et al., 2020). It is a food crop with great health care and development values. However, in China, the low yield of Tartary buckwheat seriously restricts the development of the Tartary buckwheat industry (Zhou et al., 2023).

Planting densities and nitrogen fertilizer application rates determine the growth and yield formation of crops (Zhu et al., 2023). Studies have shown that the population leaf area and dry matter accumulation of crops increased with the increase in planting density within a certain range. However, with the further increase in planting density, individual development deteriorated and population photosynthetic capacity decreased due to the competition for nutrients, water, light, and other resources among crop individuals, resulting in a decrease in final yield (Hecht et al., 2019). Therefore, planting at a reasonably high density can improve crop canopy light transmittance and increase photosynthetic effective area; these effects are conducive to the increase in yield (Chong et al., 2023). The application of nitrogen fertilizer is closely related to the formation of crop yield. However, high rates of nitrogen fertilizer application in actual production lead to a decrease in nitrogen fertilizer utilization rates and output ratios due to the one-sided emphasis that farmers place on the promotion of yield by using nitrogen fertilizer. At the same time, the ecological environment is seriously polluted due to the excessive application of nitrogen fertilizer (Ju et al., 2006; Zhang et al., 2013; Wang et al., 2016). This phenomenon is particularly common in the cultivation of Tartary buckwheat (Zhou et al., 2023). Therefore, coordinating the relationship among planting density, nitrogen fertilizer application rate, and crop yield is an important guarantee for achieving high and stable crop yields, improving crop production efficiency, and achieving safe production (Leskovsek et al., 2012).

In recent years, many scholars have conducted systematic relevant research on the above topics. Wang F. D. et al. (2020) found that for rape–early rice–late rice, increased planting density with reduced nitrogen application could meet nitrogen demand during growth, ensure normal growth, and considerably improve the utilization rate of nitrogen fertilizer to achieve or even slightly increase the yield of conventional cultivation. Deng et al. (2020) also found that high planting density with low nitrogen application could improve the morphology and distribution of rice roots and increase grain yield. Some scholars have performed related research on buckwheat. Fan et al. (2018) discovered that under treatment with an appropriate amount of nitrogen fertilizer, an excessively high or low planting density affected the yield of common buckwheat. Xiang et al. (2016) found that under the appropriate nitrogen fertilizer treatment, the yield of Tartary buckwheat increased first and then decreased with the increase in planting density. Our previous study demonstrated that compared with the Tartary buckwheat yield obtained at the conventional planting density of 10 × 10^5^ plants·ha^−1^ and nitrogen fertilizer dosage of 135 kg·ha^−1^ in Guizhou Province, that obtained with an appropriate reduction in nitrogen application (by 20%, that is, 108·ha^−1^) and increase in planting density (by 20%, that is, 12 × 10^5^ plants·ha^−1^) had increased by 17.39% (Zhang et al., 2021b). To date, a large number of studies with nitrogen fertilizer and planting density have focused on the yield of buckwheat. However, little is known about application of nitrogen fertilizer and planting density on the physiological characteristics of buckwheat. Given that Tartary buckwheat has a low fertilizer demand (Zhou et al., 2023), we hypothesized that replacing the application of nitrogen fertilizer with increased planting density may affect the formation of final yield by regulating the root and shoot growth, leaf photosynthetic capacity, and senescence of Tartary buckwheat. However, studies relevant to this hypothesis are lacking. Therefore, in this work, the Tartary buckwheat variety ‘Jinqiao 2’ (JQ2), which has a large planting area in Guizhou Province, was used as the experimental material, and the effects of treatments with different planting densities on the growth, senescence, and yield formation of Tartary buckwheat without nitrogen fertilization were studied. The major objective was to reveal the effect of planting density on the senescence and yield of Tartary buckwheat under low nitrogen conditions. The results provide a novel agronomic method for the high-yielding and nitrogen-saving cultivation of Tartary buckwheat.

## 2 Materials and methods

### 2.1 Plant materials and growth

JQ2 (local main cultivar of Tartary buckwheat in Guizhou Province, China) was provided by the School of Life Science of Guizhou Normal University, China. The experiment was conducted during the Tartary buckwheat growing season (August–November) from 2020 to 2021 at Xiaba’s Cultivation Experiment Station of Guizhou Normal University, Guiyang City, Guizhou Province, China (106.94°E, 26.73°N). The soil used was yellow loam with 19.98 mg·kg^−1^ available phosphorus, 24.92 mg·kg^−1^ available potassium, 8.18 mg·kg^−1^ ammonium nitrogen, and 28.16‰ organic matter.

The experiment was performed by using a single-factor randomized block design with three replicates. Seeds were sown on August 29, 2020 and August 28, 2021. Strip-seeding sowing was adopted and the experimental plot area was 10 m^2^ (5m long, 2m wide, 6 rows, 33 cm row spacing). Five density treatments in the absence of nitrogen fertilizer were established, namely, 8 × 10^5^ (80 plants·m^−2^, 133 plants per row), 10 × 10^5^ (100 plants·m^−2^, 167 plants per row), 12 × 10^5^ (120 plants·m^−2^, 200 plants per row), 14 × 10^5^ (140 plants·m^−2^, 233 plants per row), and 16 × 10^5^ plants·ha^−1^ (160 plants·m^−2^, 267 plants per row), which were denoted as D1, D2, D3, D4, and D5, respectively. In accordance with the local optimal dosage, 70 kg·ha^−1^ phosphate fertilizer (calcium superphosphate, containing 14% P_2_O_5_) and 5.0 kg·ha^−1^ potassium fertilizer (potassium chloride, containing 60% K_2_O) were mixed and applied as the base fertilizer at one time (Zhang et al., 2021c). No fertilizer was applied during the whole growth period. Grains were harvested on November 23, 2020 and November 20, 2021 when 70% of the grains had matured. At the seedling stage, seedlings were thinned or supplemented to maintain the base seedling number of each plot at the set value of the experiment. Artificial irrigation was performed in accordance with the principle of extreme drought and thorough irrigation. Other field management and pest control practices were consistent with those performed for local high-yielding cultivation (Zhang et al., 2021a). The monthly average temperatures, sunshine, and rainfall from August to November were 17.0˚C, 118.2h, and 93.6mm in 2020, respectively, and 21.1˚C, 147.4h, and 112.1 mm in 2021, respectively.

### 2.2 Sample preparation

At the seedling, flowering, grain filling, and mature stages, approximately 10 Tartary buckwheat plants from each plot with uniform growth were sampled with complete roots. The roots were rinsed with running water and cut off after the water was filtered. Five of the roots were used to determine root morphology indices, and the other five were used to determine root activity. Leaves on nodes 1–3 at the top of the main stem were sampled, treated with liquid nitrogen for 30 s, and then stored in a refrigerator at −80°C. They were used to determine the contents of chlorophyll a, chlorophyll b, carotene, and malondialdehyde (MDA) and the activities of peroxidase (POD) and superoxide dismutase (SOD).

### 2.3 Measurement

In accordance with the method of Guo et al. (2022), the number of main stem nodes, main stem branches, leaves, and grains per plant; weight of grains per plant; and 1000-grain weight were determined. One square meter at the center of each plot (not sampled during the experiment and excluding border plants) was randomly selected, and the grains of all Tartary buckwheat plants were collected to determine yield after air drying.

A root scanning analysis system (GXY-A, Zhejiang Tuopu Instrument) was used to quantify the length, surface area, volume, and mean diameter of Tartary buckwheat roots (Zhang et al., 2023b). Root activity was determined through the 2,3,5-triphenyl tetrazolium chloride method (Li, 2000).

The contents of chlorophyll a, chlorophyll b, and carotenoids in the leaves during each period were measured in accordance with the method of Li (2000).

The net photosynthetic rate, transpiration rate, stomatal conductance, and intercellular CO_2_ concentration of the leaves on nodes 1–3 at the top of the main stem at seedling, flowering, grain filling, and mature stages were determined by using an LI-COR-6400 portable photosynthetic apparatus (Li-Cor 6400, Li-Cor, Lincoln, NE, USA). The assay time was from 10:00 a.m. to 11:00 a.m., and 10 leaves were measured for each treatment (Zhang et al., 2021c).

SOD activity in leaves was determined via the NBT reduction method. POD activity was determined through the guaiacol method. MDA content was determined by using the thiobarbituric acid method (Li, 2000).

### 2.4 Statistical analysis

Data were processed by using Microsoft Excel 2003 and SPSS 22.0. One-way ANOVA was performed, and means were compared by using the least significant difference at the 0.05 probability level. The results of 2020 and 2021 were similar. Therefore, the data were presented as the average across the two study years, and the data of 2020 (**Supplementary Table S1**-**Table S6**) and 2021 (**Supplementary Table S7**-**Table S12**) were deposited as **Supplementary Data**.

## 3 Results

### 3.1 Effects of plant density on the agronomic traits of Tartary buckwheat

**Figure 1:**
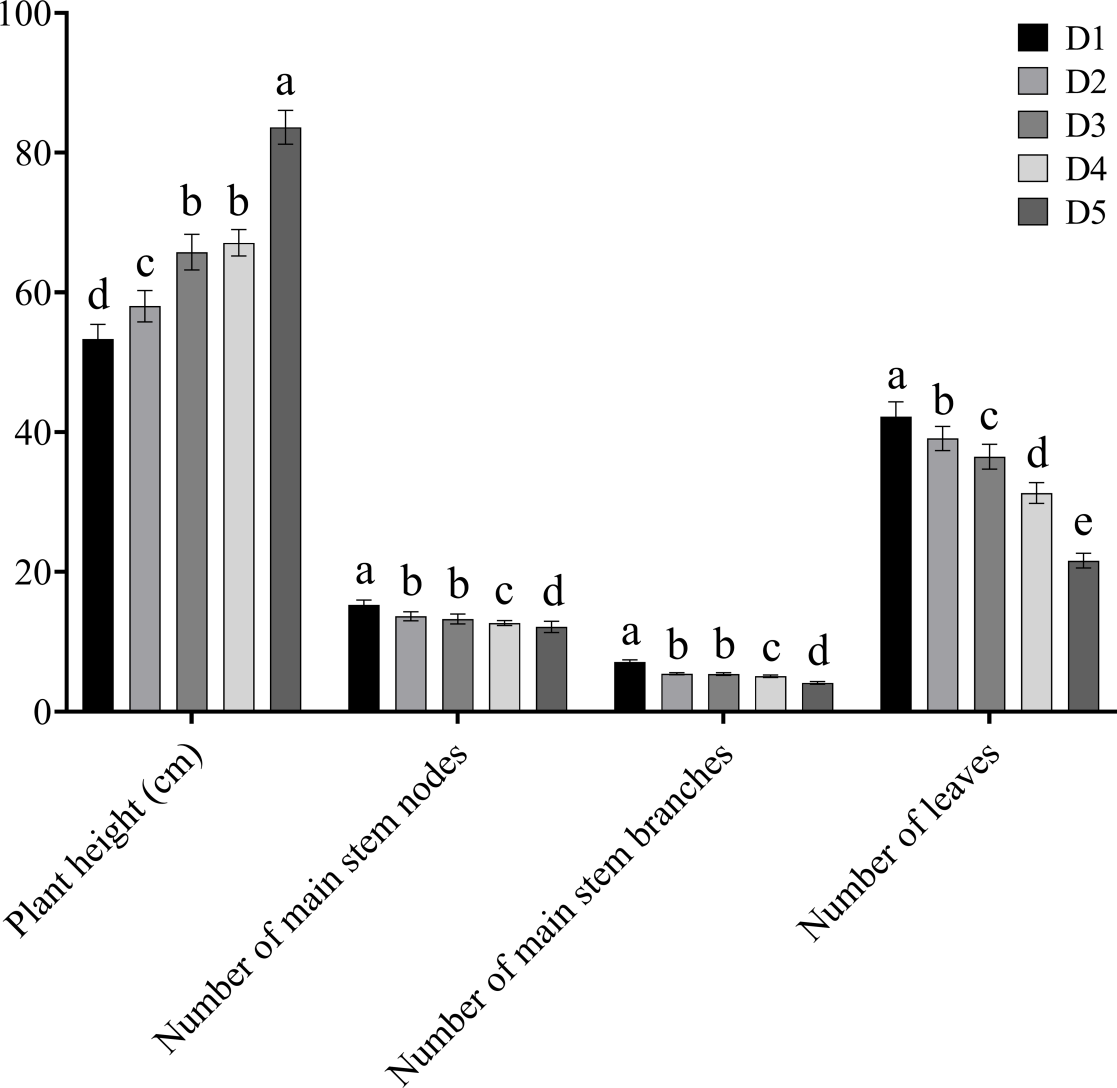
Effects of different planting density treatments on agronomic traits of Tartary buckwheat. Data are presented as mean ± standard error of the mean. Small letter in the same column means significant difference at *p* < 0.05. D1: the planting density was 8×10^5^ plants·ha^-1^; D2: the planting density was 10×10^5^ plants·ha^-1^; D3: the planting density was 12×10^5^ plants·ha^-1^; D4: the planting density was 14×10^5^ plants·ha^-1^; D5: the planting density was 16×10^5^ plants·ha^-1^.

The plant height of Tartary buckwheat continuously increased with the increase in planting density and was significantly higher under the D5 treatment than under the other four treatments (**Figure 1**). The numbers of nodes on the main stem, branches on the main stem, and leaves decreased continuously with the increase in planting density and were significantly higher under the D1 treatment than under the other four treatments.

### 3.2 Effects of plant density on the yield of Tartary buckwheat

**Figure 2:**
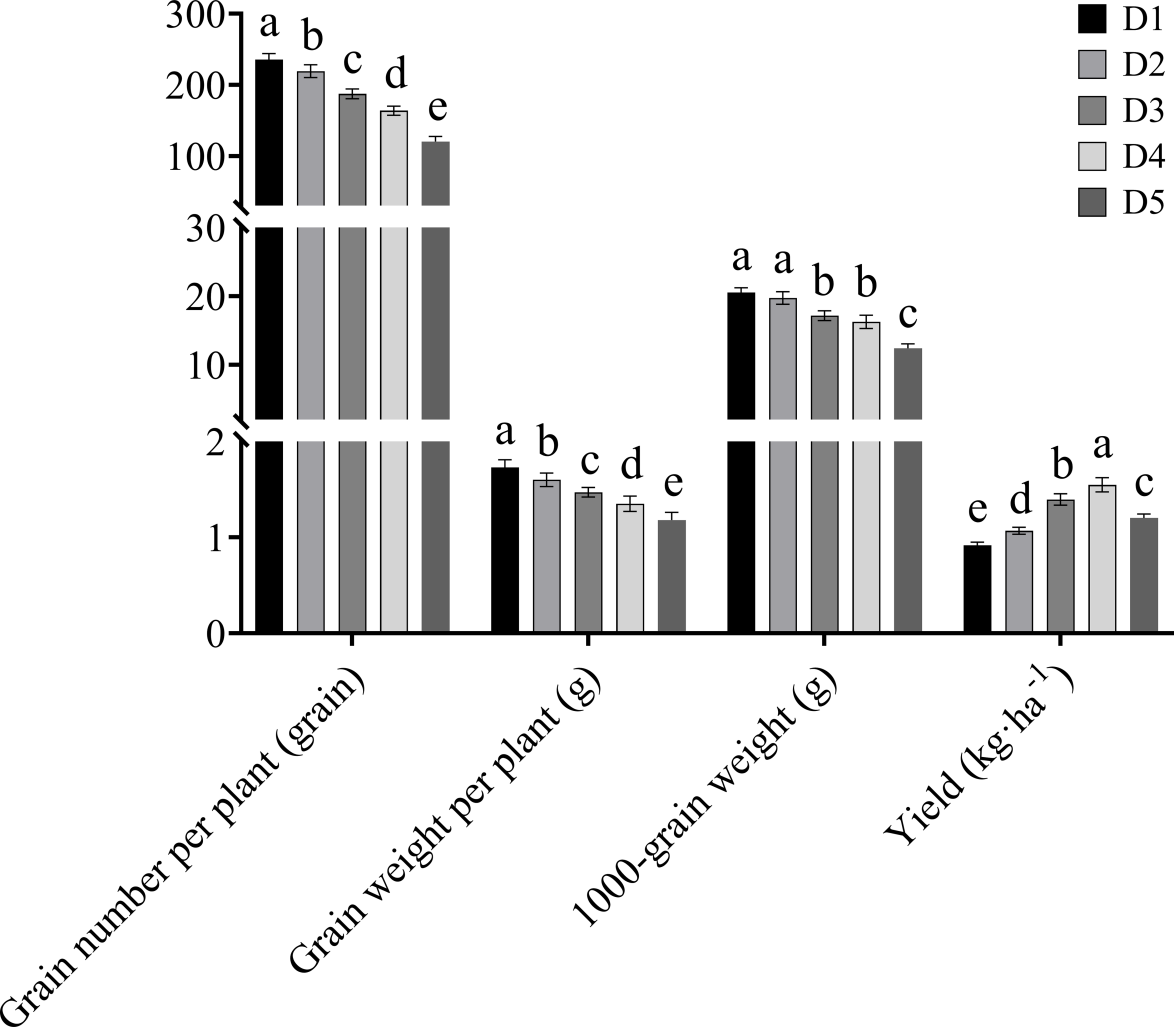
Effects of different planting density treatments on yield of Tartary buckwheat. Data are presented as mean ± standard error of the mean. Small letter in the same column means significant difference at *p* < 0.05. D1: the planting density was 8×10^5^ plants·ha^-1^; D2: the planting density was 10×10^5^ plants·ha^-1^; D3: the planting density was 12×10^5^ plants·ha^-1^; D4: the planting density was 14×10^5^ plants·ha^-1^; D5: the planting density was 16×10^5^ plants·ha^-1^.

The grain number per plant, grain weight per plant, and 1000-grain weight of Tartary buckwheat decreased continuously with the increase in planting density (**Figure 2**). The yield of Tartary buckwheat increased first and then decreased with the increase in planting density. Compared with the D1, D2, D3 and, D5 treatments, the D4 treatment increased yield by 68.61%, 44.82%, 11.00%, and 22.36%, respectively.

### 3.3 Effects of plant density on the root morphology and activity of Tartary buckwheat

**Figure 3:**
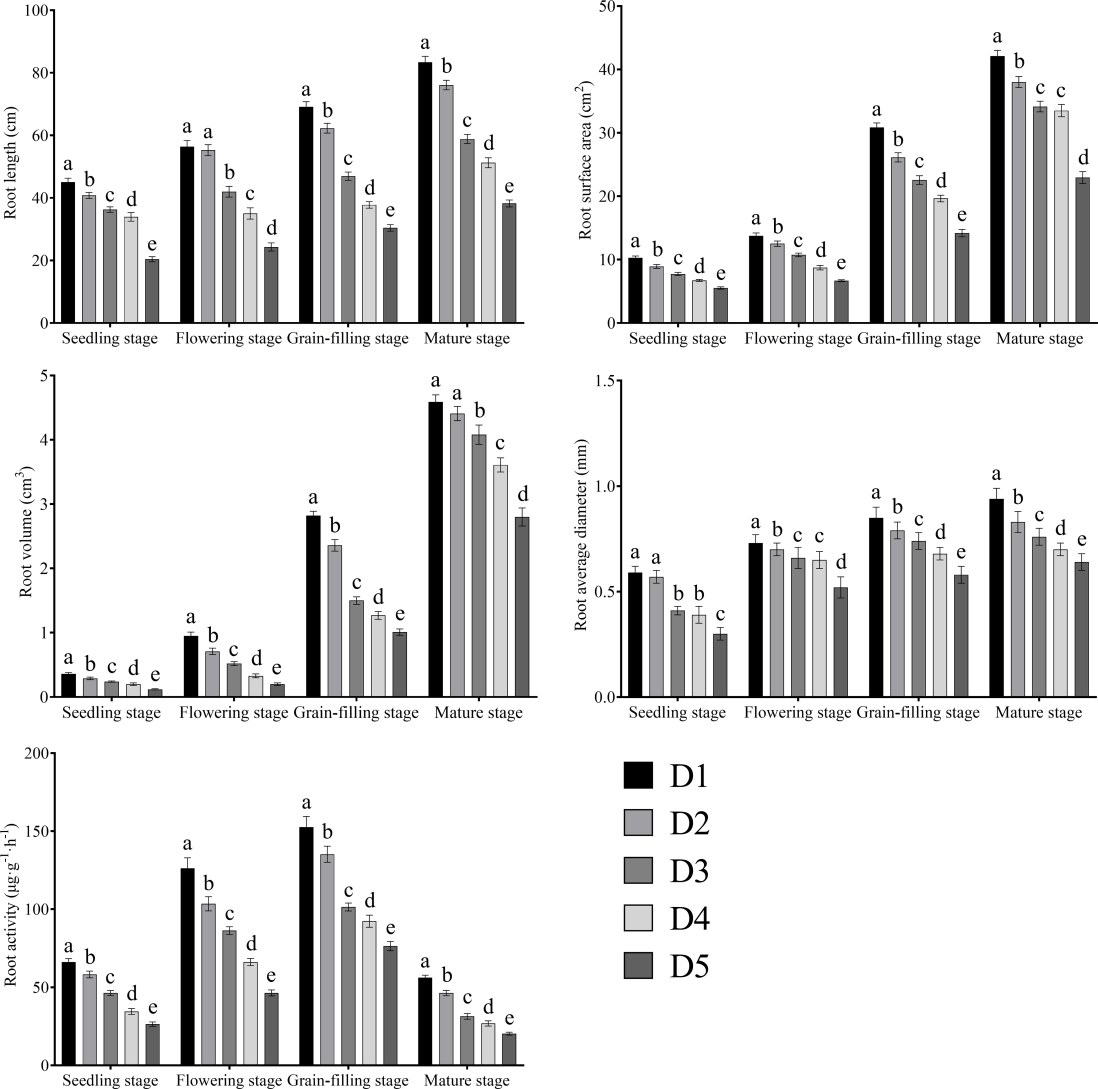
Effects of different planting density treatments on root morphology and root activity of Tartary buckwheat. Data are presented as mean ± standard error of the mean. Small letter in the same column means significant difference at *p* < 0.05. D1: the planting density was 8×10^5^ plants·ha^-1^; D2: the planting density was 10×10^5^ plants·ha^-1^; D3: the planting density was 12×10^5^ plants·ha^-1^; D4: the planting density was 14×10^5^ plants·ha^-1^; D5: the planting density was 16×10^5^ plants·ha^-1^.

The root length, surface area, volume, and average diameter of Tartary buckwheat increased continuously with the advancement of growth (**Figure 3**). Root activity increased first and then decreased with the advancement of growth and reached the maximum at the grain filling stage. Root length, surface area, volume, average diameter, and activity continuously declined with the increase in planting density and were highest under the D1 treatment and lowest under the D5 treatment.

### 3.4 Effects of plant density on the chlorophyll content of Tartary buckwheat

**Figure 4:**
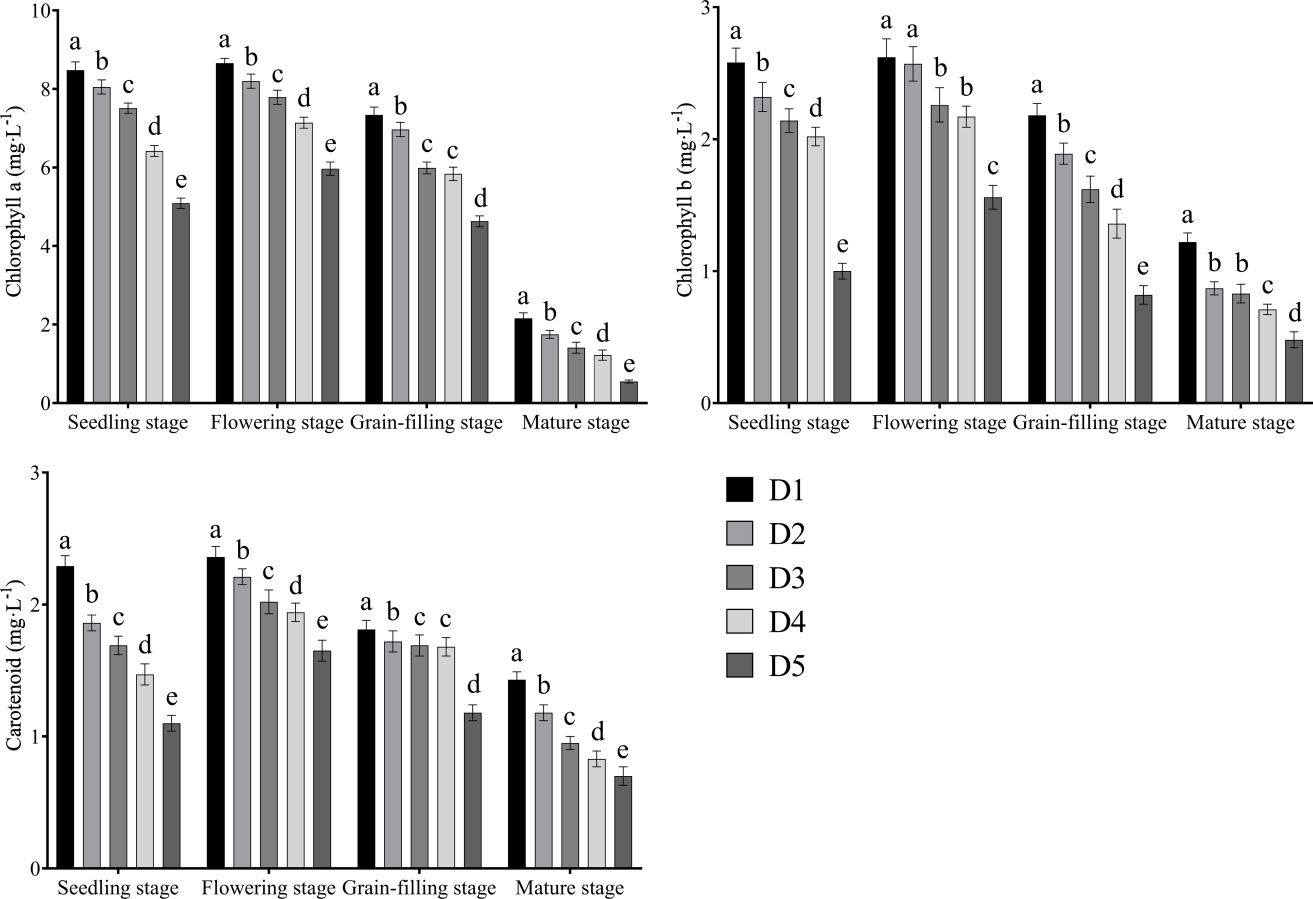
Effects of different planting density treatments on chlorophyll content of Tartary buckwheat. Data are presented as mean ± standard error of the mean. Small letter in the same column means significant difference at *p* < 0.05. D1: the planting density was 8×10^5^ plants·ha^-1^; D2: the planting density was 10×10^5^ plants·ha^-1^; D3: the planting density was 12×10^5^ plants·ha^-1^; D4: the planting density was 14×10^5^ plants·ha^-1^; D5: the planting density was 16×10^5^ plants·ha^-1^.

The contents of chlorophyll a, chlorophyll b, and carotenoids increased first and then decreased with the advancement of growth and reached their maximum values at the flowering stage (**Figure 4**). The contents of chlorophyll a, chlorophyll b, and carotenoids decreased continuously with the increase in planting density.

### 3.5 Effects of plant density on the photosynthetic characteristics of Tartary buckwheat

**Figure 5:**
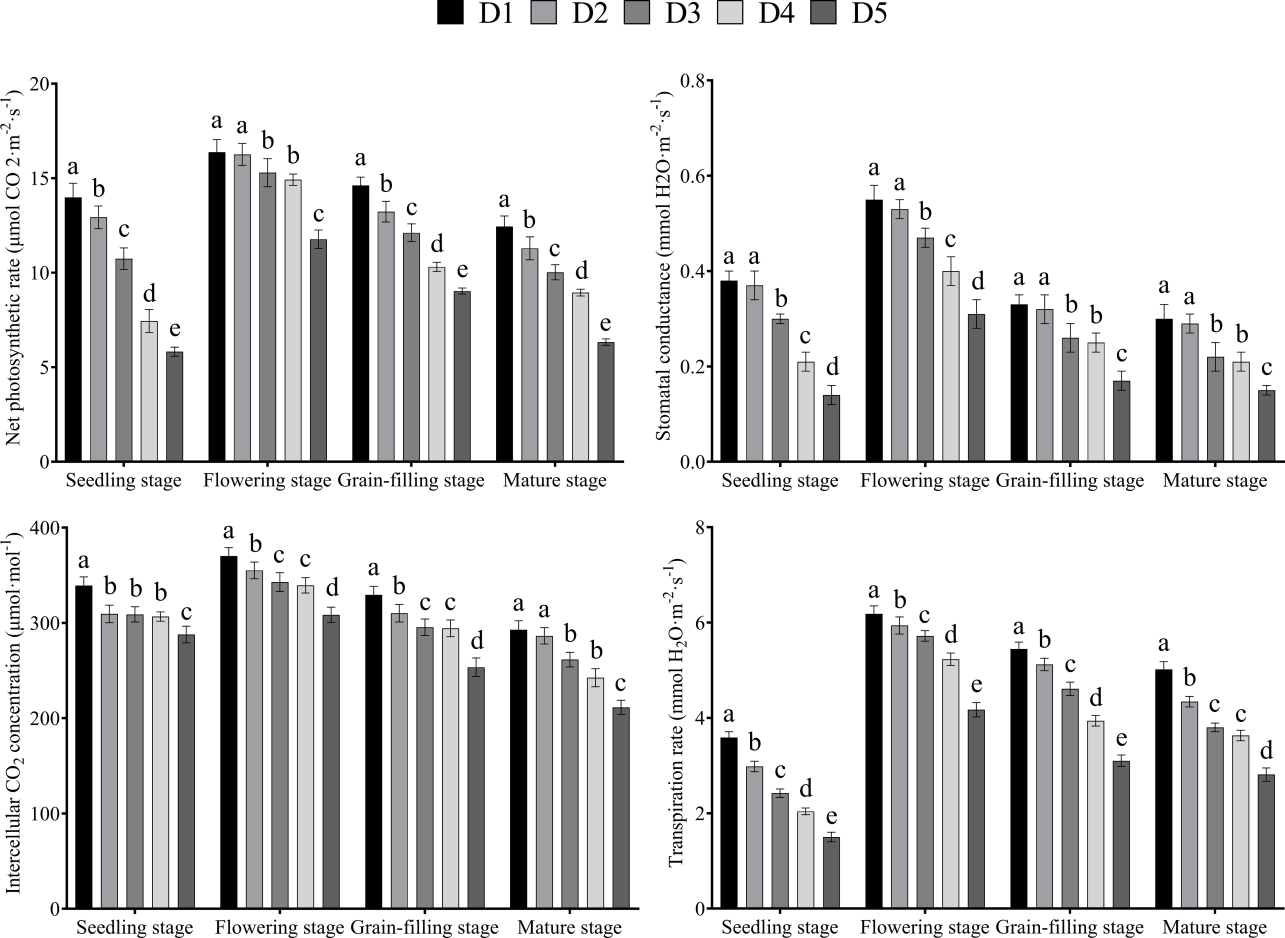
Effects of different planting density treatment on photosynthetic characteristics of Tartary buckwheat. Data are presented as mean ± standard error of the mean. Small letter in the same column means significant difference at *p* < 0.05. D1: the planting density was 8×10^5^ plants·ha^-1^; D2: the planting density was 10×10^5^ plants·ha^-1^; D3: the planting density was 12×10^5^ plants·ha^-1^; D4: the planting density was 14×10^5^ plants·ha^-1^; D5: the planting density was 16×10^5^ plants·ha^-1^.

The net photosynthetic rate, transpiration rate, intercellular CO_2_ concentration, and transpiration rate of leaves first increased and then decreased with the advancement of growth and reached their maximum values at the flowering stage (**Figure 5**). The net photosynthetic rate, transpiration rate, intercellular CO_2_ concentration, and transpiration rate of leaves decreased continuously with the increase in planting density.

### 3.6 Effects of plant density on the antioxidant enzyme activity and MDA content of Tartary buckwheat

**Figure 6:**
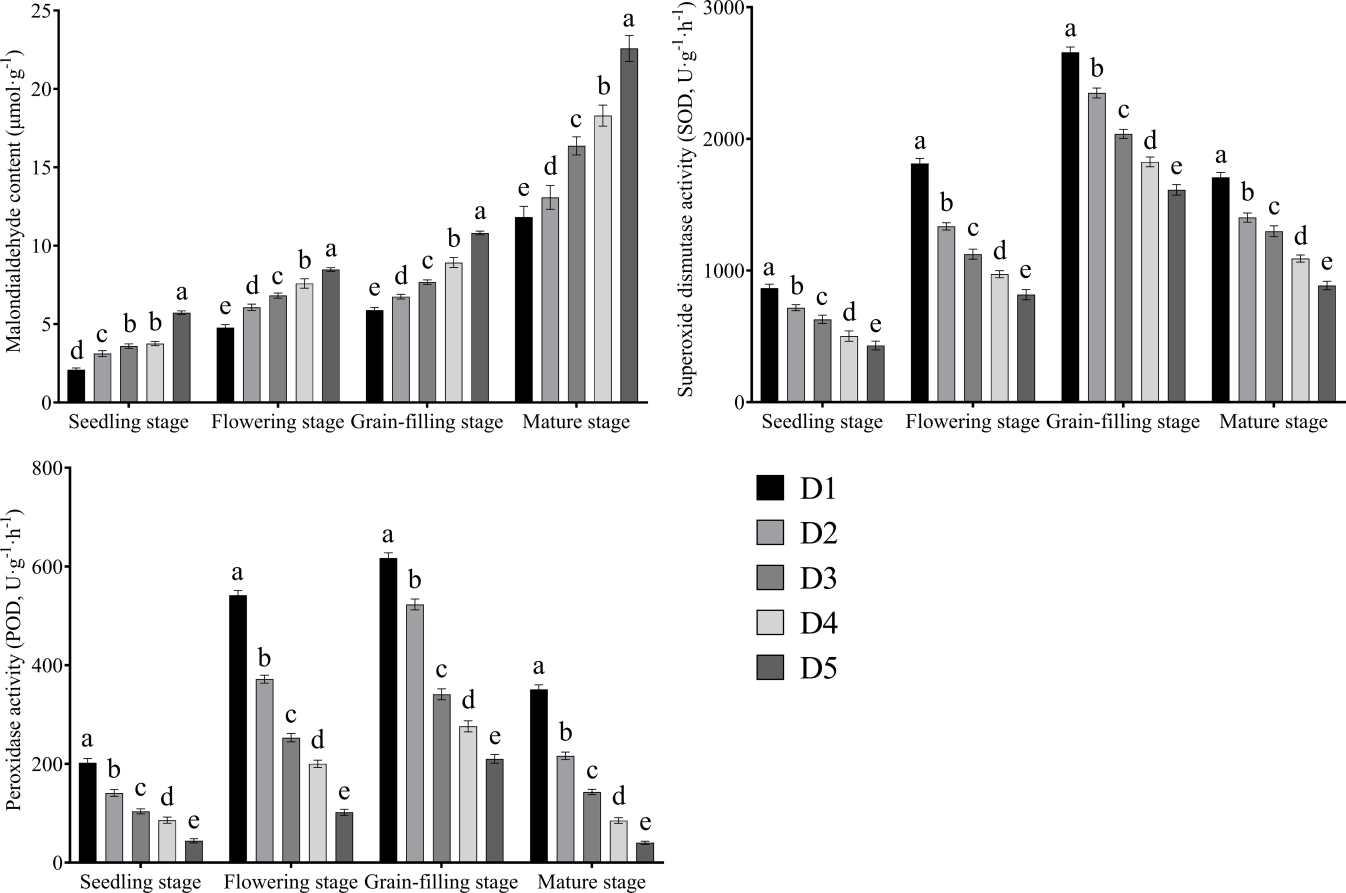
Effects of different planting density treatment on antioxidant enzyme activities and malondialdehyde content of Tartary buckwheat. Data are presented as mean ± standard error of the mean. Small letter in the same column means significant difference at *p* < 0.05. D1: the planting density was 8×10^5^ plants·ha^-1^; D2: the planting density was 10×10^5^ plants·ha^-1^; D3: the planting density was 12×10^5^ plants·ha^-1^; D4: the planting density was 14×10^5^ plants·ha^-1^; D5: the planting density was 16×10^5^ plants·ha^-1^.

The MDA content of leaves increased continuously with the advancement of growth (**Figure 6**). SOD and POD activities first increased and then decreased with growth and reached their maximum at the grain filling stage. The MDA content of leaves increased continuously with the increase in planting density. SOD and POD activities decreased continuously with the increase in planting density and were highest under the D1 treatment and lowest under the D5 treatment.

## 4 Discussion

### 4.1 Increase in planting density aggravated the senescence of Tartary buckwheat under low nitrogen treatment

Senescence can cause the yellowing of crop leaves, severe shortening of the functional period, and cessation of photosynthesis; these effects, in turn, seriously affect crop growth and final yield formation (Abeledo et al., 2020). The activities of antioxidant enzymes, such as SOD and POD, in crop leaves affect the speed of senescence. High antioxidant enzyme activity is indicative of the strong ability of crops to scavenge intracellular reactive oxygen species and slow senescence (Wang et al., 2021). MDA is a product of the lipid peroxidation of the plant cell membrane, and its content is closely related to the speed of crop senescence (Chen et al., 2018). Zhang et al. (2023a) found that in Tartary buckwheat, the activities of SOD and POD in the leaves increased and the content of MDA decreased after the removal of apical dominance, which delayed senescence and increased grain weight and final yield. Wu et al. (2020) discovered that increasing SOD and POD activities and reducing MDA content in Tartary buckwheat leaves could delay senescence and increase yield. Stress, such as continuous cropping obstacles, lead to reductions in SOD and POD activities in buckwheat, breaking the dynamic balance between the production and scavenging of reactive oxygen species in the plant body such that reactive oxygen species accumulate, resulting in membrane damage or destruction, increasing MDA content, and accelerating senescence, thereby reducing yield (Wang F. D. et al., 2020; Zhang et al., 2021a). Nitrogen is one of the most important macroelements limiting crop growth (Sakuraba, 2022). Nitrogen deficiency causes premature leaf senescence, which leads to the accelerated maturation of the whole plant and seriously reduces crop yield (Fan et al., 2023). Zhang et al. (2021c) found that in Tartary buckwheat, low nitrogen treatment accelerated senescence, which in turn affected final yield formation. Studies have demonstrated that the root activity of crops affects the senescence of aboveground leaves, and leaf senescence can be delayed by appropriately increasing the root activity of crops (Zhou et al., 2023). Mu et al. (2018) found that low planting density could effectively alleviate the senescence of Tartary buckwheat leaves, whereas high planting density accelerated senescence. Inconsistent with the results of Mu et al. (2018), our findings showed that the activities of SOD and POD; contents of chlorophyll a, chlorophyll b, and carotenoids; and root activity of Tartary buckwheat decreased continuously with the increase in planting density, whereas MDA content increased continuously. These trends indicated that the increase in planting density would accelerate the senescence of Tartary buckwheat under low nitrogen treatment. It may be that this experiment was carried out in the absence of nitrogen fertilization and the insufficient nitrogen absorption of Tartary buckwheat, which led to the senescence of Tartary buckwheat even at low planting densities. It may also be correlated with the nutritional imbalance among nitrogen, phosphorus, and potassium in rhizosphere soil caused by absence of nitrogen fertilization.

### 4.2 Increase in planting density increased the population yield of Tartary buckwheat under low nitrogen treatment

The root is an organ for nutrient absorption by crops and has an important role in plant growth. At the same time, the strength of root activity is greatly important for the ability of crops to absorb nutrients and the measurement of senescence (Wu et al., 2020). Zhang et al. (2023c) found that increasing planting density could increase competition for nutrients among roots, thereby reducing root volume. The study of Xiang et al. (2016) on Tartary buckwheat further confirmed this conclusion, that is, with the increase in planting density, the related root indices decreased and root growth was inhibited. Consistent with the results of Zhang et al. (2023c) and Xiang et al. (2016), those of our study demonstrated that the root length, surface area, volume, average diameter, and activity of Tartary buckwheat decreased continuously with the increase in planting density. These phenomena likely occurred because under the condition of nitrogen deficiency, the increase in planting density aggravates the competition for nutrients in rhizosphere soil among Tartary buckwheat plants and inhibits root growth, which further affects the absorption of nutrients and water in rhizosphere soil by Tartary buckwheat roots, transport of dry matter, and formation of yield per plant.

Photosynthesis, as an important process of nutrient synthesis and dry matter accumulation, has a crucial effect on crop yield formation. Zhang et al. (2021) found that in cotton, net photosynthetic rate, respiration rate, and stomatal conductance increased first and then decreased with the increase in density. Inconsistent with the above results but similar to the results of Ju et al. (2022), our findings showed that the net photosynthetic rate, respiration rate, stomatal conductance, and intercellular CO_2_ concentration decreased continuously with the increase in planting density in the absence of nitrogen fertilizer. This difference may be related to the fact that this experiment was performed in the absence of nitrogen fertilizer. The increase in density led to the increase in the Tartary buckwheat population and the intensification of the competition among individuals for nutrients, water, light, and other resources. Such competition led to the decrease in plant individual photosynthetic capacity and finally affected the accumulation of photosynthetic assimilates, resulting in the decrease in the indices of yield per plant, such as grain number per plant, grain weight per plant, and 1000-grain weight. In addition, studies have shown that the assimilates produced by the upper and middle leaves on the main stem of buckwheat have the highest contribution to grain filling (Zhang et al., 2023a). Therefore, the photosynthetic capacity of these leaves determines the yield per plant of buckwheat. The senescence of the upper and middle leaves cause leaf yellowing and decrease photosynthetic capacity, resulting in insufficient filling materials in the later grain filling stage and ultimately in poor grain filling degree. This effect may be another important reason for the considerable decrease in grain weight per plant, grain number per plant, and 1000-grain weight of Tartary buckwheat with the increase in planting density.

Planting density is a key factor affecting crop yield. It not only affects the light interception rate of the crop canopy and the ventilation of the population, it also affects the accumulation and distribution of crop photosynthetic products, thus affecting the final yield (Cong et al., 2023). Fan et al. (2018) showed that the yield of common buckwheat increased first and then decreased with the increase in planting density. Wu et al. (2023) obtained similar results. Consistent with the results of the above study and of the research of Ju et al. (2022) on oats, the findings of our experiment revealed that the yield of JQ2 increased first and then decreased with the increase in planting density in the absence of nitrogen fertilizer. This might be due to the weak light irradiance to the leaf by shading and strong competition among individuals with the increasing plant density (Fan et al., 2018). The number of grains per plant and 1000-grain weight are the key factors affecting yield per plant in the yield composition of Tartary buckwheat (Zhang et al., 2023a). The results of our experiment showed that inconsistent with population yield, the yield per plant of Tartary buckwheat continuously declined with the increase in planting density. This finding showed that the increase in the population yield of the Tartary buckwheat under low nitrogen treatment is mainly achieved by the increase in the number of plants per unit area. At the same time, obtaining high population yields under low nitrogen treatment rely on maximizing planting density.

Notably, the yield of JQ2 in Guizhou Province is generally 1510 kg·ha^−1^ (Zhang et al., 2021c). In this experiment, when the planting density increased to 14 × 10^5^ plants·ha^−1^ in the absence of nitrogen fertilizer, the yield of JQ2 reached 1548.26 kg·ha^−1^, which was approximately 38 kg·ha^−1^ higher than the average yield. This increase may be related to the low fertilizer demand of Tartary buckwheat. At the same time, compared with the local conventional planting density of 10 × 10^5^ plants·ha^−1^ and the amount of nitrogen fertilizer of 135 kg·ha^−1^, the planting density in our experiment increased by 4 × 10^5^ plants·ha^−1^ (approximately 20 kg·ha^−1^) in the absence of nitrogen fertilizer and the population yield increased by approximately 38 kg·ha^−1^. Overall, in this experiment, 135 kg·ha^−1^ nitrogen fertilizer was saved and Tartary buckwheat yield increased by 18 kg·ha^−1^ (representing an increase of 38 kg·ha^−1^ in yield reduced 20 kg·ha^−1^ in sowing amount increased). The prices of nitrogen fertilizer and Tartary buckwheat in Guizhou Province were $2.0 and $1.37·kg^−1^, respectively. Therefore, the production cost of $270·ha^−1^ can be saved and benefit increased by $24.66·ha^−1^. The total of those two terms is $ 294.66·ha^−1^, indicating that costs greatly reduced and benefit improved. This finding indicates that the high-yielding and nitrogen-saving production of Tartary buckwheat can be achieved by controlling nitrogen with density. This technology is worth using in production.

## 5 Conclusions

An increase in planting density could reduce the SOD and POD activities, root activity, and chlorophyll content and increase MDA content in the leaves of Tartary buckwheat, thus accelerating the senescence of Tartary buckwheat under low nitrogen treatment. Under low nitrogen treatment, the population yield of the Tartary buckwheat increased mainly through the increase in the number of plants per unit area. A threshold for planting density for increasing the population yield of Tartary buckwheat under low nitrogen treatment exists, that is, 14 × 10^5^ plants·ha^−1^. The high-yielding and nitrogen-saving cultivation of Tartary buckwheat, which has low fertilizer demand, can be achieved by controlling nitrogen with density in production.

## Data availability statement

The original contributions presented in the study are included in the article/**Supplementary Material**. Further inquiries can be directed to the corresponding authors.

## Author contributions

QZ: Investigation, Methodology, Writing – original draft, Writing – review & editing. PH: Data curation, Methodology, Writing – original draft. JT: Data curation, Methodology, Software, Writing – original draft. KH: Conceptualization, Funding acquisition, Investigation, Methodology, Writing – original draft, Writing – review & editing. XH: Writing – review & editing.
