# Supplementary material for: Increasing planting density can improve the yield of Tartary buckwheat
Source: Front Plant Sci. 2023 Dec 13;14:1313181. doi: 10.3389/fpls.2023.1313181 (PMC10753774; doi:10.3389/fpls.2023.1313181)
Supplement: Supplementary file 1 [file Table_1.docx]

**Supplementary data in 2020**

TABLE S1 Effects of different planting density treatments on agronomic traits of Tartary buckwheat

| Treatment | Plant height (cm) | Number of main stem nodes | Number of main stem branches | Number of leaves |
| --- | --- | --- | --- | --- |
| D1 | 54.63±2.27d | 14.24±0.62a | 7.85±0.34a | 37.9±1.98a |
| D2 | 57.36±2.15c | 13.13±0.75b | 5.71±0.17b | 36.0±1.49ab |
| D3 | 60.74±2.59b | 12.58±0.74c | 5.67±0.20b | 34.3±1.93b |
| D4 | 59.03±1.82b | 12.13±0.38c | 5.48±0.17b | 29.4±1.88c |
| D5 | 85.19±2.88a | 11.61±0.99d | 4.24±0.22c | 20.7±1.33d |

Data are presented as mean ± standard error of the mean. Small letter in the same column means significant

difference at *p* < 0.05. D1: the planting density was 8×10^5^ plants·ha^-1^; D2: the planting density was 10×10^5^ plants·ha^-1^; D3: the planting density was 12×10^5^ plants·ha^-1^; D4: the planting density was 14×10^5^ plants·ha^-1^; D5: the planting density was 16×10^5^ plants·ha^-1^.

TABLE S2 Effects of different planting density treatments on yield of Tartary buckwheat

| Treatment | Grain number per plant(grain) | Grain weight per plant (g) | 1000-grain weight  (g) | Yield  (kg·ha^-1^) |
| --- | --- | --- | --- | --- |
| D1 | 224.77±7.47a | 1.53±0.06a | 19.13±0.56a | 908.54±36.29e |
| D2 | 215.05±8.41a | 1.48±0.06a | 17.87±0.92b | 1013.1±26.89d |
| D3 | 178.02±6.78b | 1.31±0.03b | 16.09±0.88c | 1377.39±61.71b |
| D4 | 172.88±4.62b | 1.19±0.07c | 14.59±0.97d | 1498.96±70.39a |
| D5 | 128.30±7.03c | 1.09±0.08d | 11.34±0.86e | 1143.64±31.28c |

Data are presented as mean ± standard error of the mean. Small letter in the same column means significant

difference at *p* < 0.05. D1: the planting density was 8×10^5^ plants·ha^-1^; D2: the planting density was 10×10^5^ plants·ha^-1^; D3: the planting density was 12×10^5^ plants·ha^-1^; D4: the planting density was 14×10^5^ plants·ha^-1^; D5: the planting density was 16×10^5^ plants·ha^-1^.

TABLE S3 Effects of different planting density treatments on root morphology and root activity of Tartary buckwheat

| Item | Treatment | Period | | | |
| --- | --- | --- | --- | --- | --- |
|  |  | Seedling stage | Flowering stage | Grain-filling stage | Mature stage |
| Root length (cm) | D1 | 43.16±1.48a | 46.45±1.22a | 58.54±1.56a | 74.34±1.91a |
|  | D2 | 38.14±0.82b | 46.06±1.14a | 52.33±1.94b | 72.03±1.80a |
|  | D3 | 34.26±0.88c | 31.81±1.29b | 33.75±1.50c | 46.43±1.92b |
|  | D4 | 33.35±1.77c | 26.15±0.87c | 23.47±1.02d | 33.40±1.29c |
|  | D5 | 17.00±0.79d | 17.11±0.91d | 17.59±1.08e | 28.12±0.88d |
| Root surface area (cm^2^) | D1 | 9.00±0.21a | 9.33±0.18a | 22.56±0.78a | 38.32±0.98a |
|  | D2 | 8.27±0.26a | 7.06±0.14b | 20.01±0.63b | 37.46±0.72a |
|  | D3 | 7.07±0.15b | 6.98±0.19b | 19.04±0.68c | 34.06±0.84b |
|  | D4 | 6.25±0.12b | 6.52±0.10b | 18.66±0.61c | 33.77±0.98b |
|  | D5 | 5.14±0.12c | 5.69±0.11c | 12.93±0.85d | 22.23±0.86c |
| Root volume (cm^3^) | D1 | 0.25±0.01a | 0.94±0.05a | 2.81±0.09a | 4.20±0.10a |
|  | D2 | 0.20±0.02b | 0.60±0.04b | 2.57±0.08a | 4.19±0.11a |
|  | D3 | 0.16±0.01c | 0.40±0.02bc | 1.24±0.05b | 3.79±0.14b |
|  | D4 | 0.13±0.01c | 0.23±0.02c | 1.06±0.04bc | 3.42±0.08c |
|  | D5 | 0.08±0.01d | 0.08±0.01d | 0.90±0.04c | 2.44±0.13d |
| Root average diameter (mm) | D1 | 0.59±0.03a | 0.73±0.03a | 0.85±0.04a | 0.94±0.05a |
|  | D2 | 0.57±0.03a | 0.70±0.02a | 0.79±0.03b | 0.83±0.05b |
|  | D3 | 0.46±0.03b | 0.66±0.04b | 0.74±0.03c | 0.76±0.04c |
|  | D4 | 0.39±0.02c | 0.65±0.03b | 0.68±0.05d | 0.70±0.04d |
|  | D5 | 0.30±0.02d | 0.52±0.04c | 0.58±0.04e | 0.64±0.03e |
| Root activity  (μg·g^-1^·h^-1^) | D1 | 57.38±2.06a | 118.95±6.60a | 143.32±3.80a | 45.59±1.31a |
|  | D2 | 46.79±2.42b | 94.58±3.56b | 122.40±5.58b | 40.26±1.57b |
|  | D3 | 39.54±1.40c | 81.43±2.91c | 80.17±1.85c | 21.14±1.60c |
|  | D4 | 27.35±1.66d | 57.40±2.19d | 77.77±2.08c | 19.45±1.39c |
|  | D5 | 22.48±1.43e | 40.42±1.77e | 66.53±1.95d | 17.22±0.75d |

Data are presented as mean ± standard error of the mean. Small letter in the same column means significant

difference at *p* < 0.05. D1: the planting density was 8×10^5^ plants·ha^-1^; D2: the planting density was 10×10^5^ plants·ha^-1^; D3: the planting density was 12×10^5^ plants·ha^-1^; D4: the planting density was 14×10^5^ plants·ha^-1^; D5: the planting density was 16×10^5^ plants·ha^-1^.

TABLE S4 Effects of different planting density treatments on chlorophyll content of Tartary buckwheat

| Item | Treatment | Period | | | |
| --- | --- | --- | --- | --- | --- |
|  |  | Seedling stage | Flowering stage | Grain-filling stage | Mature stage |
| Chlorophyll a  (mg⋅L^-1^) | D1 | 8.20±0.28a | 8.50±0.13a | 7.06±0.19a | 1.56±0.14a |
|  | D2 | 8.07±0.18a | 8.05±0.16b | 6.89±0.18a | 1.47±0.12a |
|  | D3 | 7.60±0.10b | 7.76±0.21b | 6.01±0.14b | 1.29±0.17b |
|  | D4 | 6.21±0.13c | 7.10±0.10c | 5.80±0.13b | 1.23±0.11b |
|  | D5 | 4.36±0.15d | 5.41±0.17d | 4.13±0.12c | 0.25±0.02c |
| Chlorophyll b  (mg⋅L^-1^) | D1 | 2.30±0.09a | 2.32±0.12a | 1.51±0.09a | 0.94±0.06a |
|  | D2 | 2.25±0.08b | 2.30±0.13a | 1.37±0.07b | 0.62±0.04b |
|  | D3 | 2.21±0.09b | 2.17±0.14b | 1.35±0.08bc | 0.61±0.08b |
|  | D4 | 2.19±0.07b | 2.11±0.07b | 1.33±0.09c | 0.59±0.05b |
|  | D5 | 0.82±0.06c | 1.37±0.10c | 0.71±0.06d | 0.39±0.06c |
| Carotenoid  (mg⋅L^-1^) | D1 | 1.90±0.07a | 1.88±0.06a | 1.74±0.06a | 1.18±0.06a |
|  | D2 | 1.71±0.06b | 1.86±0.07a | 1.69±0.08b | 1.00±0.05b |
|  | D3 | 1.55±0.08c | 1.81±0.09b | 1.68±0.07b | 0.87±0.06c |
|  | D4 | 1.35±0.07d | 1.76±0.08c | 1.67±0.08b | 0.81±0.05c |
|  | D5 | 1.00±0.08e | 1.59±0.07d | 1.10±0.05c | 0.69±0.07d |

Data are presented as mean ± standard error of the mean. Small letter in the same column means significant

difference at *p* < 0.05. D1: the planting density was 8×10^5^ plants·ha^-1^; D2: the planting density was 10×10^5^ plants·ha^-1^; D3: the planting density was 12×10^5^ plants·ha^-1^; D4: the planting density was 14×10^5^ plants·ha^-1^; D5: the planting density was 16×10^5^ plants·ha^-1^.

TABLE S5 Effects of different planting density treatment on photosynthetic characteristics of Tartary buckwheat

| Item | Treatment | Period | | | |
| --- | --- | --- | --- | --- | --- |
|  |  | Seedling stage | Flowering stage | Grain-filling stage | Mature stage |
| Net photosynthetic rate (μmol CO _2_·m^-2^·s^-1^) | D1 | 12.97±0.68a | 14.37±0.79a | 13.26±0.28a | 11.52±0.44a |
|  | D2 | 12.30±0.63a | 14.31±0.52a | 12.27±0.59b | 10.35±0.67b |
|  | D3 | 8.81±0.75b | 13.56±0.87b | 10.98±0.37c | 8.87±0.30c |
|  | D4 | 4.68±0.10c | 13.38±0.34b | 8.96±0.05d | 8.67±0.15c |
|  | D5 | 3.05±0.13d | 10.36±0.58c | 7.53±0.07e | 4.80±0.11d |
| Stomatal conductance (mmol H_2_O·m^-2^·s^-1^) | D1 | 0.35±0.02a | 0.49±0.02a | 0.20±0.01a | 0.21±0.03a |
|  | D2 | 0.35±0.03a | 0.47±0.03a | 0.19±0.02a | 0.21±0.02a |
|  | D3 | 0.28±0.02b | 0.42±0.02b | 0.19±0.03a | 0.16±0.03b |
|  | D4 | 0.18±0.03c | 0.34±0.03c | 0.18±0.02a | 0.15±0.02b |
|  | D5 | 0.11±0.01d | 0.27±0.02d | 0.14±0.02b | 0.13±0.01c |
| Intercellular CO_2_ concentration (μmol·mol^-1^) | D1 | 320.78±8.76a | 343.54±8.913a | 309.10±9.56a | 276.62±9.50a |
|  | D2 | 294.61±9.27b | 330.81±8.23b | 294.14±7.79a | 267.12±9.29a |
|  | D3 | 294.22±9.90b | 329.71±9.24b | 283.34±9.81b | 247.35±9.21b |
|  | D4 | 292.54±7.03b | 327.95±8.42b | 282.31±9.52b | 235.52±8.05c |
|  | D5 | 284.88±8.62b | 306.74±9.09c | 239.99±8.15c | 201.49±8.17d |
| Transpiration rate  (mmol H_2_O·m^-2^·s^-1^) | D1 | 3.31±0.08a | 5.60±0.14a | 5.08±0.16a | 4.57±0.15a |
|  | D2 | 2.52±0.09b | 5.49±0.18ab | 4.77±0.12a | 3.76±0.09b |
|  | D3 | 1.82±0.07c | 5.41±0.08b | 4.18±0.15b | 3.37±0.12c |
|  | D4 | 1.32±0.05d | 4.95±0.16c | 2.90±0.11c | 3.10±0.09c |
|  | D5 | 0.52±0.06e | 3.17±0.15d | 2.53±0.09d | 2.65±0.13d |

Data are presented as mean ± standard error of the mean. Small letter in the same column means significant

difference at *p* < 0.05. D1: the planting density was 8×10^5^ plants·ha^-1^; D2: the planting density was 10×10^5^ plants·ha^-1^; D3: the planting density was 12×10^5^ plants·ha^-1^; D4: the planting density was 14×10^5^ plants·ha^-1^; D5: the planting density was 16×10^5^ plants·ha^-1^.

TABLE S6 Effects of different planting density treatment on antioxidant enzyme activities and malondialdehyde content of Tartary buckwheat

| Item | Treatment | Period | | | |
| --- | --- | --- | --- | --- | --- |
|  |  | Seedling stage | Flowering stage | Grain-filling stage | Mature stage |
| Malondialdehyde content  (μmol·g^-1^) | D1 | 2.32±0.11d | 5.05±0.21e | 5.92±0.19e | 12.90±0.55e |
|  | D2 | 3.50±0.18c | 6.31±0.29d | 7.09±0.17d | 14.11±0.91d |
|  | D3 | 3.98±0.17b | 7.17±0.16c | 8.06±0.14c | 17.13±0.62c |
|  | D4 | 4.17±0.14b | 7.92±0.39b | 9.57±0.41b | 19.10±0.47b |
|  | D5 | 6.05±0.12a | 8.72±0.11a | 11.80±0.52a | 24.43±0.89a |
| Superoxide dismutase activity (SOD, U·g^-1^·h^-1^) | D1 | 861.52±27.98a | 1680.62±41.25a | 2473.15±39.53a | 1526.50±26.29a |
|  | D2 | 617.05±16.82b | 1221.25±11.57b | 2282.48±43.57b | 1294.03±36.17b |
|  | D3 | 556.72±34.35c | 1004.71±31.44c | 1863.61±40.98c | 1280.23±35.88b |
|  | D4 | 454.13±41.77d | 920.62±29.95d | 1710.98±37.24d | 1063.63±37.29c |
|  | D5 | 397.13±29.68e | 668.88±37.43e | 1485.78±36.86e | 773.49±48.67d |
| Peroxidase activity (POD, U·g^-1^·h^-1^) | D1 | 180.15±7.94a | 479.85±8.60a | 514.11±9.48a | 329.22±9.82a |
|  | D2 | 105.60±4.37b | 251.71±7.68b | 430.87±11.59b | 148.01±7.78b |
|  | D3 | 64.31±2.21c | 176.76±8.62c | 245.40±9.93c | 114.04±5.79c |
|  | D4 | 55.25±2.89c | 146.57±7.05d | 221.66±9.32c | 63.89±4.88d |
|  | D5 | 20.52±1.60d | 76.39±2.07e | 148.63±8.06d | 37.35±2.83e |

Data are presented as mean ± standard error of the mean. Small letter in the same column means significant

difference at *p* < 0.05. D1: the planting density was 8×10^5^ plants·ha^-1^; D2: the planting density was 10×10^5^ plants·ha^-1^; D3: the planting density was 12×10^5^ plants·ha^-1^; D4: the planting density was 14×10^5^ plants·ha^-1^; D5: the planting density was 16×10^5^ plants·ha^-1^.

**Supplementary data in 2021**

TABLE S7 Effects of different planting density treatments on agronomic traits of Tartary buckwheat

| Treatment | Plant height (cm) | Number of main stem nodes | Number of main stem branches | Number of leaves |
| --- | --- | --- | --- | --- |
| D1 | 52.03±1.91e | 16.38±0.69a | 6.39±0.27a | 46.5±2.29a |
| D2 | 58.70±2.33d | 14.17±0.55b | 5.13±0.12b | 42.2±1.97b |
| D3 | 70.80±2.51c | 13.96±0.68b | 5.07±0.18b | 38.7±1.60c |
| D4 | 75.17±1.96b | 13.23±0.34c | 4.68±0.20c | 33.2±1.08d |
| D5 | 82.07±1.95a | 12.67±0.62d | 4.02±0.15d | 22.5±0.77e |

Data are presented as mean ± standard error of the mean. Small letter in the same column means significant

difference at *p* < 0.05. D1: the planting density was 8×10^5^ plants·ha^-1^; D2: the planting density was 10×10^5^ plants·ha^-1^; D3: the planting density was 12×10^5^ plants·ha^-1^; D4: the planting density was 14×10^5^ plants·ha^-1^; D5: the planting density was 16×10^5^ plants·ha^-1^.

TABLE S8 Effects of different planting density treatments on yield of Tartary buckwheat

| Treatment | Grain number per plant(grain) | Grain weight per plant (g) | 1000-grain weight  (g) | Yield  (kg·ha^-1^) |
| --- | --- | --- | --- | --- |
| D1 | 246.23±8.98a | 1.93±0.09a | 21.97±0.76a | 927.98±27.75e |
| D2 | 223.15±9.79b | 1.72±0.07b | 21.61±0.92a | 1125.06±46.81d |
| D3 | 196.48±6.86c | 1.63±0.06c | 18.21±0.54b | 1412.45±57.21b |
| D4 | 154.30±8.07d | 1.51±0.09d | 17.93±0.98b | 1597.56±78.61a |
| D5 | 112.26±7.02e | 1.27±0.08e | 13.46±0.41c | 1260.44±54.77c |

Data are presented as mean ± standard error of the mean. Small letter in the same column means significant

difference at *p* < 0.05. D1: the planting density was 8×10^5^ plants·ha^-1^; D2: the planting density was 10×10^5^ plants·ha^-1^; D3: the planting density was 12×10^5^ plants·ha^-1^; D4: the planting density was 14×10^5^ plants·ha^-1^; D5: the planting density was 16×10^5^ plants·ha^-1^.

TABLE S9 Effects of different planting density treatments on root morphology and root activity of Tartary buckwheat

| Item | Treatment | Period | | | |
| --- | --- | --- | --- | --- | --- |
|  |  | Seedling stage | Flowering stage | Grain-filling stage | Mature stage |
| Root length (cm) | D1 | 46.90±1.09a | 66.31±2.77a | 79.64±1.76a | 92.48±1.80a |
|  | D2 | 43.44±1.08b | 64.50±2.36a | 72.25±1.20b | 80.19±1.24b |
|  | D3 | 38.26±0.88c | 52.13±2.12b | 60.17±1.15c | 71.27±1.02c |
|  | D4 | 34.53±0.98d | 43.95±2.72c | 52.09±1.07d | 69.10±1.86c |
|  | D5 | 23.82±0.80e | 31.53±1.68d | 43.25±1.02e | 48.36±1.34d |
| Root surface area (cm^2^) | D1 | 11.60±0.35a | 18.21±0.69a | 39.16±0.64a | 45.94±0.79a |
|  | D2 | 9.57±0.36b | 17.98±0.71a | 32.27±0.81b | 38.58±0.99b |
|  | D3 | 8.43±0.35c | 14.52±0.37b | 26.08±0.69c | 34.24±0.83c |
|  | D4 | 7.21±0.18d | 10.96±0.60c | 20.64±0.43c | 33.25±0.86c |
|  | D5 | 5.92±0.24e | 7.67±0.18d | 15.43±0.30d | 23.67±0.98d |
| Root volume (cm^3^) | D1 | 0.47±0.02a | 0.96±0.07a | 2.83±0.04a | 4.98±0.12a |
|  | D2 | 0.38±0.02b | 0.82±0.06b | 2.15±0.09b | 4.63±0.10b |
|  | D3 | 0.32±0.01c | 0.64±0.03c | 1.76±0.07c | 4.37±0.16c |
|  | D4 | 0.27±0.02d | 0.43±0.03d | 1.48±0.07d | 3.80±0.13d |
|  | D5 | 0.16±0.01e | 0.32±0.02e | 1.12±0.06e | 3.16±0.15e |
| Root average diameter (mm) | D1 | 0.65±0.03a | 0.79±0.04a | 0.97±0.06a | 1.07±0.04a |
|  | D2 | 0.63±0.02a | 0.75±0.03b | 0.91±0.05b | 0.92±0.05b |
|  | D3 | 0.49±0.02b | 0.68±0.05c | 0.83±0.04c | 0.81±0.04c |
|  | D4 | 0.41±0.05c | 0.67±0.04c | 0.76±0.02d | 0.70±0.02d |
|  | D5 | 0.32±0.03d | 0.56±0.06d | 0.62±0.04e | 0.65±0.04e |
| Root activity  (μg·g^-1^·h^-1^) | D1 | 74.82±2.51a | 133.47±6.89a | 162.04±9.68a | 66.91±1.67a |
|  | D2 | 69.71±1.86b | 112.36±5.49b | 148.12±4.74b | 52.38±1.81b |
|  | D3 | 53.12±1.89c | 91.25±2.02c | 122.73±3.19c | 41.62±1.95c |
|  | D4 | 41.65±2.11d | 74.90±2.45d | 106.91±5.67d | 34.27±1.89d |
|  | D5 | 30.38±1.36e | 52.32±2.05e | 86.27±3.90e | 23.46±1.10e |

Data are presented as mean ± standard error of the mean. Small letter in the same column means significant

difference at *p* < 0.05. D1: the planting density was 8×10^5^ plants·ha^-1^; D2: the planting density was 10×10^5^ plants·ha^-1^; D3: the planting density was 12×10^5^ plants·ha^-1^; D4: the planting density was 14×10^5^ plants·ha^-1^; D5: the planting density was 16×10^5^ plants·ha^-1^.

TABLE S10 Effects of different planting density treatments on chlorophyll content of Tartary buckwheat

| Item | Treatment | Period | | | |
| --- | --- | --- | --- | --- | --- |
|  |  | Seedling stage | Flowering stage | Grain-filling stage | Mature stage |
| Chlorophyll a  (mg⋅L^-1^) | D1 | 8.76±0.14a | 8.82±0.11a | 7.62±0.20a | 2.76±0.14a |
|  | D2 | 8.03±0.18b | 8.35±0.19b | 7.05±0.18b | 2.03±0.08b |
|  | D3 | 7.42±0.16c | 7.82±0.14c | 5.97±0.16c | 1.53±0.11c |
|  | D4 | 6.63±0.15d | 7.18±0.17d | 5.88±0.21c | 1.21±0.15d |
|  | D5 | 5.82±0.11e | 6.53±0.16e | 5.13±0.16d | 0.85±0.06e |
| Chlorophyll b  (mg⋅L^-1^) | D1 | 2.86±0.12a | 2.92±0.16a | 2.85±0.09a | 1.50±0.08a |
|  | D2 | 2.39±0.14b | 2.84±0.13b | 2.41±0.10b | 1.12±0.06b |
|  | D3 | 2.07±0.09c | 2.35±0.11c | 1.89±0.12c | 1.05±0.05c |
|  | D4 | 1.85±0.06d | 2.23±0.09d | 1.39±0.13d | 0.83±0.04d |
|  | D5 | 1.18±0.05e | 1.75±0.08e | 0.93±0.07e | 0.57±0.05e |
| Carotenoid  (mg⋅L^-1^) | D1 | 2.68±0.09a | 2.84±0.09a | 1.88±0.07a | 1.68±0.06a |
|  | D2 | 2.01±0.06b | 2.56±0.05b | 1.75±0.08b | 1.36±0.07b |
|  | D3 | 1.83±0.095c | 2.23±0.09c | 1.70±0.09c | 1.03±0.05c |
|  | D4 | 1.59±0.08d | 2.12±0.07d | 1.69±0.06c | 0.85±0.07d |
|  | D5 | 1.20±0.03e | 1.71±0.08e | 1.26±0.07d | 0.71±0.06e |

Data are presented as mean ± standard error of the mean. Small letter in the same column means significant

difference at *p* < 0.05. D1: the planting density was 8×10^5^ plants·ha^-1^; D2: the planting density was 10×10^5^ plants·ha^-1^; D3: the planting density was 12×10^5^ plants·ha^-1^; D4: the planting density was 14×10^5^ plants·ha^-1^; D5: the planting density was 16×10^5^ plants·ha^-1^.

TABLE S11 Effects of different planting density treatment on photosynthetic characteristics of Tartary buckwheat

| Item | Treatment | Period | | | |
| --- | --- | --- | --- | --- | --- |
|  |  | Seedling stage | Flowering stage | Grain-filling stage | Mature stage |
| Net photosynthetic rate (μmol CO _2_·m^-2^·s^-1^) | D1 | 14.99±0.81a | 18.39±0.52a | 15.98±0.59a | 13.36±0.67a |
|  | D2 | 13.56±0.57b | 18.21±0.64a | 14.19±0.51b | 12.23±0.55b |
|  | D3 | 12.67±0.39c | 17.02±0.60b | 13.24±0.56c | 11.17±0.49c |
|  | D4 | 10.20±0.52d | 16.46±0.25b | 11.66±0.42d | 9.23±0.21d |
|  | D5 | 8.59±0.35e | 13.18±0.40c | 10.53±0.25e | 7.86±0.24e |
| Stomatal conductance (mmol H_2_O·m^-2^·s^-1^) | D1 | 0.41±0.03a | 0.61±0.03a | 0.46±0.02a | 0.39±0.02a |
|  | D2 | 0.39±0.02a | 0.59±0.02a | 0.45±0.03a | 0.37±0.02a |
|  | D3 | 0.32±0.01b | 0.52±0.02b | 0.33±0.02b | 0.28±0.03b |
|  | D4 | 0.24±0.02c | 0.46±0.02c | 0.32±0.02b | 0.27±0.03b |
|  | D5 | 0.17±0.02d | 0.35±0.03d | 0.20±0.01c | 0.17±0.01c |
| Intercellular CO_2_ concentration (μmol·mol^-1^) | D1 | 357.92±9.27a | 396.92±8.67a | 349.78±8.58a | 309.20±9.19a |
|  | D2 | 324.33±9.05b | 379.43±9.18b | 326.28±10.5b | 305.84±7.72a |
|  | D3 | 323.70±6.14b | 356.05±9.62c | 307.58±7.33c | 275.71±6.14b |
|  | D4 | 320.86±2.72b | 350.89±7.31c | 306.45±7.94c | 249.38±10.93c |
|  | D5 | 290.82±8.93c | 310.18±6.92d | 266.93±11.36d | 221.23±6.87d |
| Transpiration rate  (mmol H_2_O·m^-2^·s^-1^) | D1 | 3.87±0.16a | 6.76±0.19a | 5.82±0.11a | 5.47±0.16a |
|  | D2 | 3.44±0.12b | 6.39±0.18b | 5.47±0.15b | 4.92±0.13b |
|  | D3 | 3.02±0.10c | 6.03±0.14c | 5.04±0.12c | 4.23±0.07c |
|  | D4 | 2.76±0.09d | 5.51±0.09d | 4.98±0.11c | 4.16±0.13c |
|  | D5 | 2.48±0.14e | 5.17±0.14e | 3.67±0.15d | 2.97±0.15d |

Data are presented as mean ± standard error of the mean. Small letter in the same column means significant

difference at *p* < 0.05. D1: the planting density was 8×10^5^ plants·ha^-1^; D2: the planting density was 10×10^5^ plants·ha^-1^; D3: the planting density was 12×10^5^ plants·ha^-1^; D4: the planting density was 14×10^5^ plants·ha^-1^; D5: the planting density was 16×10^5^ plants·ha^-1^.

TABLE S12 Effects of different planting density treatment on antioxidant enzyme activities and malondialdehyde content of Tartary buckwheat

| Item | Treatment | Period | | | |
| --- | --- | --- | --- | --- | --- |
|  |  | Seedling stage | Flowering stage | Grain-filling stage | Mature stage |
| Malondialdehyde content  (μmol·g^-1^) | D1 | 1.86±0.10d | 4.51±0.16e | 5.86±0.15e | 10.74±0.82e |
|  | D2 | 2.74±0.19c | 5.83±0.12d | 6.41±0.13d | 12.07±0.61d |
|  | D3 | 3.22±0.11b | 6.47±0.16c | 7.30±0.14c | 15.61±0.54c |
|  | D4 | 3.35±0.12b | 7.26±0.21b | 8.29±0.23b | 17.50±0.87b |
|  | D5 | 5.41±0.10a | 8.24±0.12a | 9.82±0.14a | 20.73±0.76a |
| Superoxide dismutase activity (SOD, U·g^-1^·h^-1^) | D1 | 871.82±30.29a | 1946.28±33.92a | 2843.51±37.83a | 1890.16±46.71a |
|  | D2 | 818.13±28.51b | 1449.13±36.76b | 2413.82±32.46b | 1509.67±34.04b |
|  | D3 | 701.42±29.10c | 1243.43±44.81c | 2210.95±27.33c | 1316.07±47.11c |
|  | D4 | 549.21±36.86d | 1024.26±24.05d | 1939.06±36.72d | 1117.85±17.64d |
|  | D5 | 464.35±37.09e | 964.46±39.90e | 1738.30±42.48e | 998.73±16.38e |
| Peroxidase activity (POD, U·g^-1^·h^-1^) | D1 | 224.15±9.90a | 604.27±9.92a | 720.09±11.74a | 372.84±8.82a |
|  | D2 | 176.92±9.59b | 491.85±8.59b | 615.41±10.29b | 284.81±7.67b |
|  | D3 | 143.47±8.22c | 369.56±8.36c | 436.70±11.91c | 172.30±5.06c |
|  | D4 | 117.03±9.69d | 254.27±7.66d | 330.86±13.11d | 106.79±6.78d |
|  | D5 | 68.18±6.94e | 127.63±10.09e | 271.93±9.63e | 42.95±3.74e |

Data are presented as mean ± standard error of the mean. Small letter in the same column means significant

difference at *p* < 0.05. D1: the planting density was 8×10^5^ plants·ha^-1^; D2: the planting density was 10×10^5^ plants·ha^-1^; D3: the planting density was 12×10^5^ plants·ha^-1^; D4: the planting density was 14×10^5^ plants·ha^-1^; D5: the planting density was 16×10^5^ plants·ha^-1^.
